# Structural and nanomechanical properties of BiFeO_3_ thin films deposited by radio frequency magnetron sputtering

**DOI:** 10.1186/1556-276X-8-297

**Published:** 2013-06-25

**Authors:** Sheng-Rui Jian, Huang-Wei Chang, Yu-Chin Tseng, Ping-Han Chen, Jenh-Yih Juang

**Affiliations:** 1 Department of Materials Science and Engineering, I-Shou University, Kaohsiung 840, Taiwan; 2 Department of Applied Physics, Tunghai University, Taichung 407, Taiwan; 3 Department of Electrophysics, National Chiao Tung University, Hsinchu 300, Taiwan

**Keywords:** BiFeO_3_ thin films, XRD, AFM, Nanoindentation, Hardness

## Abstract

The nanomechanical properties of BiFeO_3_ (BFO) thin films are subjected to nanoindentation evaluation. BFO thin films are grown on the Pt/Ti/SiO_2_/Si substrates by using radio frequency magnetron sputtering with various deposition temperatures. The structure was analyzed by X-ray diffraction, and the results confirmed the presence of BFO phases. Atomic force microscopy revealed that the average film surface roughness increased with increasing of the deposition temperature. A Berkovich nanoindenter operated with the continuous contact stiffness measurement option indicated that the hardness decreases from 10.6 to 6.8 GPa for films deposited at 350°C and 450°C, respectively. In contrast, Young's modulus for the former is 170.8 GPa as compared to a value of 131.4 GPa for the latter. The relationship between the hardness and film grain size appears to follow closely with the Hall–Petch equation.

## Background

Multiferroic materials exhibit some unique characteristics with the co-existence of at least two kinds of long-range ordering among ferroelectricity (or antiferroelectricity), ferromagnetism (or antiferromagnetism), and ferroelasticity. Single-phase compounds in which both ferromagnetism and ferroelectricity arise independently and may couple to each other to give rise to magneto-electric interactions are ideal materials for novel functional device applications but are unfortunately rare in nature [1]. BiFeO_3_ (BFO) is one of the most important multiferroic materials so far discovered, which has a ferroelectric Curie temperature of 1,103 K [2,3] and an antiferromagnetic Néel temperature of 643 K [4]. In addition to its interesting optical properties [5], strong coupling between ferroelectric and magnetic orders is observed in BFO at room temperature, making it a strong candidate for realizing room-temperature multiferroic devices [6,7]. However, while most of the researches have been concentrated on the abovementioned magneto-electric characteristics of BFO, researches on the mechanical characteristics of this prominent functional material have been largely ignored. In particular, since the mechanical properties of materials are size-dependent, the properties obtained from thin films may substantially deviate from those of the bulk material. In view of the fact that most practical applications of functional devices are fabricated with thin films, it is desirable to carry out precise measurements of the mechanical properties of BFO thin films.

Because of its high sensitivity, excellent resolution, and easy operation, nanoindentation has been widely used for characterizing the mechanical properties of various nanoscale materials [8,9] and thin films [10-12]. Among the mechanical characteristics of interest, the hardness, Young's modulus, and the elastic/plastic deformation behaviors of the interested material can be readily obtained from nanoindentation measurements. For instance, by analyzing the load–displacement curves obtained during the nanoindentation following the methods proposed by Oliver and Pharr [13], the hardness and Young's modulus of the test material can be easily obtained. In general, in order to avoid the complications arising from the substrate material, the contact depths of the indenter need to be less than 10% of the film thickness to obtain intrinsic film properties [14]. On the other hand, it is very difficult to obtain meaningful analytical results for indentation depths less than 10 nm because of the equipment limitations. Hence, for films thinner than 100 nm, it is almost impossible to obtain results without being influenced by responses from the substrate. In order to gain some insights on the substrate influences and obtain the intrinsic properties for films thinner than 100 nm, it is essential to monitor the mechanical properties as a function of depth. Herein, in this study, a continuous stiffness measurement (CSM) mode [15] was adopted to continuously monitor the hardness and Young's modulus values of BFO films as a function of the indentation depth. Variations in mechanical properties for BFO thin films deposited under different conditions are discussed in conjunction with the crystalline structure, grain size, and surface morphology of the resultant films.

## Methods

The BFO thin films investigated in this study were deposited on Pt/Ti/SiO_2_/Si(100) substrates at the deposition temperatures of 350°C, 400°C, and 450°C, respectively. The deposition process was conducted in a radio frequency magnetron sputtering system, and a commercially available Bi_1.1_FeO_3_ pellet was used as the target. The base pressure of the sputtering chamber was better than 1 × 10^−7^ Torr. During deposition, a mixed gas of Ar/O_2_ = 4:1 with a total pressure was introduced, and the input power was maintained at 80 W. All of the BFO thin films are about 200 nm thick. The composition of the film was identified by an energy-dispersive X-ray analysis and double checked by X-ray fluorescence analysis. The crystal structure of BFO thin films was analyzed by X-ray diffraction (X'Pert XRD, PANalytical B.V., Almelo, The Netherlands; CuKα, *λ* = 1.5406 Å). The surface features were examined by atomic force microscopy (AFM; Topometrix-Accures-II, Topometrix Corporation, Santa Clara, CA, USA). The root mean square of the surface roughness, *R*_RMS_, was calculated by the following equation [16]:

(1) $$R_{\mathrm{RMS}}=\sqrt{\frac{1}{N}\sum_{n=1}^{N}r_{n}^{2}}$$

Here *N* is the number of data and *r*_*n*_ is the surface height of the *n*th datum.

Nanoindentation experiments were preformed on a MTS Nano Indenter® XP system (MTS Nano Instruments, Knoxville, TN, USA) with a three-sided pyramidal Berkovich indenter tip by using the CSM technique [15]. This technique is accomplished by imposing a small, sinusoidal varying force on top of the applied linear force that drives the motion of the indenter. The displacement response of the indenter at the excitation frequency and the phase angle between the force and displacement are measured continuously as a function of the penetration depth. Solving for the in-phase and out-of-phase portions of the displacement response gives rise to the determination of the contact stiffness as a continuous function of depth. As such, the mechanical properties changing with respect to the indentation depth can be obtained. The nanoindentation measurements were carried out as follows: First, prior to applying loading on BFO thin films, nanoindentation was conducted on the standard fused silica sample to obtain the reasonable range (Young's modulus of fused silica is 68~72 GPa). Then, a constant strain rate of 0.05 s^−1^ was maintained during the increment of load until the indenter reached a depth of 60 nm into the surface. The load was then held at the maximum value of loading for 10 s in order to avoid the creep which might significantly affect the unloading behavior. The indenter was then withdrawn from the surface at the same rate until the loading has reduced to 10% of the maximum load. Then, the indenter was completely removed from the material. In this study, constant strain rate was chosen in order to avoid the strain-hardening effects. At least 20 indentations were performed on each sample, and the distance between the adjacent indents was kept at least 10 μm apart to avoid interaction.

In nanoindentation tests, the hardness is defined as the applied indentation load divided by the projected contact area as follows:

(2) $$H=\frac{P_{\max}}{A_{p}}$$

where *A*_p_ is the projected contact area between the indenter and the sample surface at the maximum indentation load, *P*_max_. For a perfectly sharp Berkovich indenter, the projected area *A*_p_ is given by $A_{p}=24.56h_{c}^{2}$ with *h*_c_ being the true contact depth.

The elastic modulus of the sample can be calculated based on the relationships developed by Sneddon [17]: $S=2\beta \;E_{r}\sqrt{A_{p}}/\sqrt{\pi }$. Here *S* is the contact stiffness of the material, and *β* is a geometric constant with *β* = 1.00 for the Berkovich indenter, respectively. The reduced elastic modulus, *E*_r_, can be calculated from the following equation:

(3) $$\frac{1}{E_{r}}=\frac{1-v_{\mathrm{film}}^{2}}{E_{\mathrm{film}}}+\frac{1-v_{i}^{2}}{E_{i}}$$

Here *v* is Poisson's ratio, and the subscripts i and f denote the parameters for the indenter and the BFO thin films, respectively. For the diamond indenter tip, *E*_i_ = 1,141 GPa and *v*_i_ = 0.07, and *v*_film_ = 0.25 is assumed for BFO thin films in this work. It is generally accepted that the indentation depth should never exceed 30% of the film thickness to avoid the substrate effect on hardness and modulus measurements [18]. Our samples and test methodology were considered as adequate based on this concept. In addition, because of the fact that it enters as $\left(1-v_{\mathrm{film}}^{2}\right)$ in the calculation of *E*, an error in the estimation of Poisson's ratio does not produce a significant effect on the resulting value of the elastic modulus of thin films [19].

## Results and discussion

**Figure 1:**
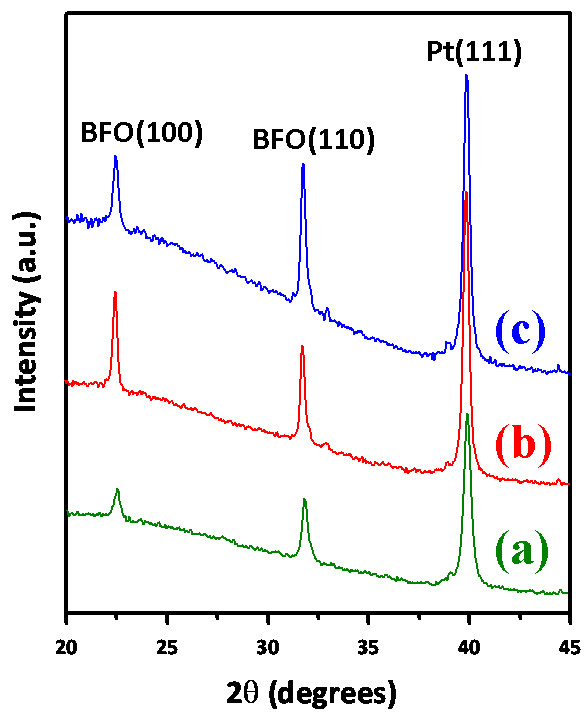
**XRD patterns of BFO thin films deposited at various deposition temperatures.** (**a**) 350°C, (**b**) 400°C, and (**c**) 450°C.

Figure 1 shows the XRD results of BFO thin films obtained with deposition temperatures of 350°C, 400°C, and 450°C, respectively. It is evident that the intensity and the full width at half maximum (FWHM) of the BFO(110) diffraction peak are both improved with the increasing deposition temperature, indicating a tendency of better film crystallinity and increased grain size. The grain size, *D*, can be estimated according to Scherrer's equation [20]:

(4) $$D=\frac{0.9\lambda }{B\cos\theta }$$

where *λ*, *B*, and *θ* are the X-ray wavelength, the FWHM of the BFO(110) diffraction peak, and the corresponding Bragg's diffraction angle, respectively. The estimated grain sizes for BFO thin films deposited at 350°C, 400°C, and 450°C are 24.5, 30.6, and 51.2 nm, respectively. As can be seen below, consistent results were obtained from the AFM examinations.

**Figure 2:**
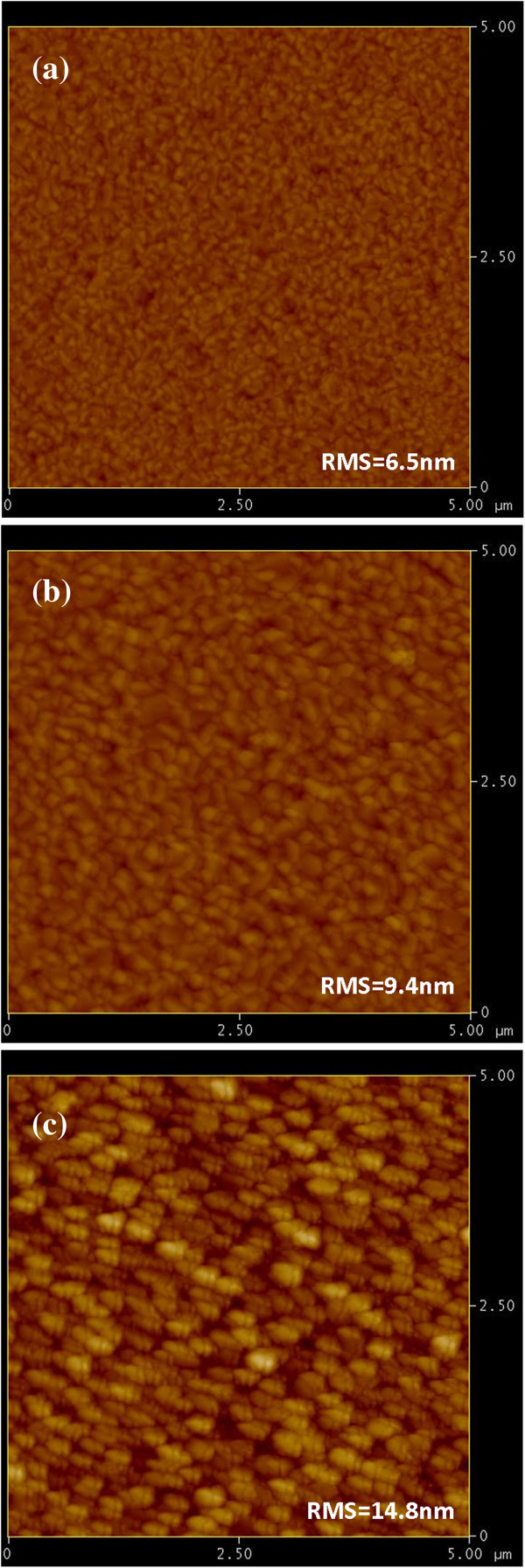
**AFM images of BFO thin films deposited at various deposition temperatures.** (**a**) 350°C, (**b**) 400°C, and (**c**) 450°C, respectively.

As shown in Figure 2, the AFM observations reveal that the *R*_RMS_ values for BFO thin films deposited at 350°C, 400°C, and 450°C are 6.5, 9.4, and 14.8 nm, respectively. Moreover, as shown in Figure 2a,b,c, the BFO thin films all exhibit similar dense, homogeneous microstructures, albeit that the grain size appears to increase with increasing deposition temperature. The average grain size obtained from image analysis on the AFM images indeed gave consistent results with those obtained from XRD analyses. Namely, the microstructure of BFO films are polycrystalline, and the grain size increases from about 24.5 nm for thin films deposited at 350°C to about 51.2 nm for thin films deposited at 450°C. This is attributed to the additional thermal energy acquired from higher deposition temperature, which may further facilitate the coalescence of the adjacent grains (or nuclei) and result in larger grains during deposition process.

**Figure 3:**
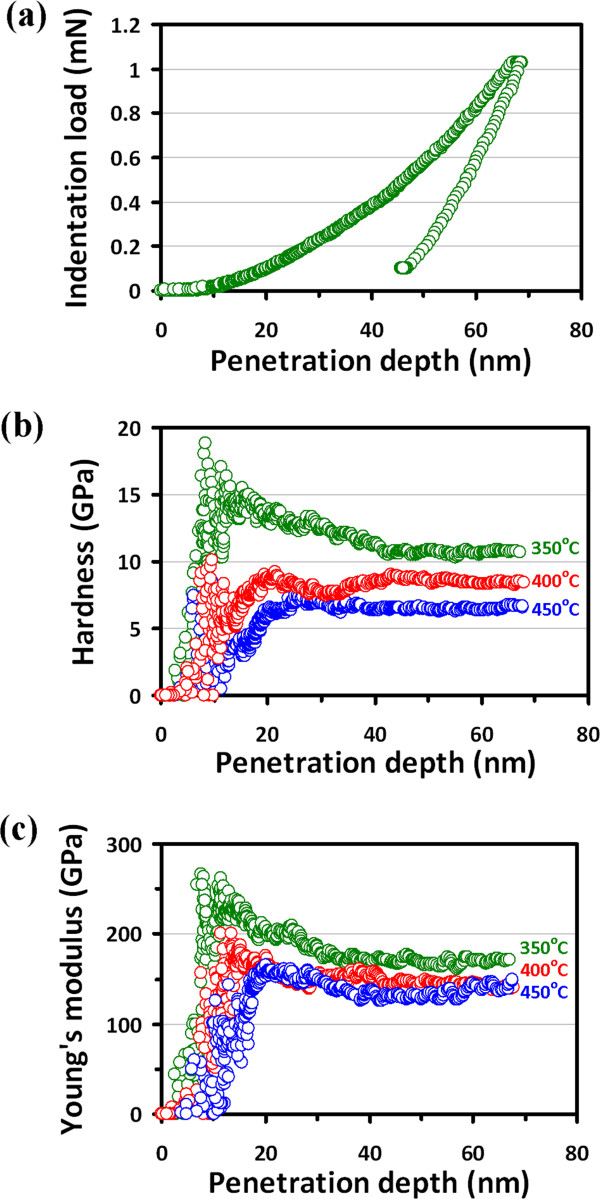
**Nanoindentation results.** (**a**) A typical load-displacement curve for BFO thin films deposited at 350°C. (**b**) The hardness-displacement curves. (**c**) Young's modulus-displacement curves for BFO thin films deposited at various deposition temperatures.

Figure 3a displays the typical load–displacement (*P*-*h*) curves for the BFO film deposited at 350°C, which reflects the general deformation behavior during the penetration of a Berkovich indenter loaded with the CSM mode. The *P*-*h* response obtained by nanoindentation contains information about the elastic behavior and plastic deformation and thus can be regarded as the ‘fingerprint’ of the properties of BFO thin films. The curve appears to be smooth and regular. The absence of any discontinuities along either the loading or unloading segment is in sharp contrast to those observed in GaN thin films [21,22] and in single-crystal Si [23,24], indicating that neither twinning nor pressure-induced phase transformation is involved here.

Figure 3b,c presents the hardness and Young's modulus versus penetration depth curves for the BFO film deposited at 350°C, 400°C, and 450°C, respectively. The curves can be roughly divided into two stages, namely, an initial increase to a maximum value followed by a subsequent decrease to a constant value. The initial sharp increase in hardness at a small penetration depth is usually attributed to the transition from purely elastic to elastic/plastic contact. Only under the condition of a fully developed plastic zone does the mean contact pressure represent the hardness. When there is no plastic zone, or only a partially formed plastic zone, the mean contact pressure measured according to the Oliver and Pharr method [13] is usually smaller than the nominal hardness. After the first stage, the hardness decreases in a rather meandering manner, presumably involving massive dislocation and grain boundary activities relevant to the fine grain structure of the films. Nevertheless, the fact that it eventually reaches a constant value at a moderate indentation depth indicates that a single material is being measured. The hardness values obtained at this stage, thus, can be regarded as the intrinsic properties of the films. The penetration depth dependence of Young's modulus (Figure 3c) behaves similarly as that of the hardness. Consequently, both mechanical parameters were determined using the curves obtained from the CSM loading scheme (Figure 3b,c) by taking the average values within the penetration depth of 40 to 60 nm. This range of penetration depth was chosen intentionally to be deep enough for observing plastic deformation during indentation yet to be shallow enough to avoid the complications arising from the effects of surface roughness [25] and substrate [18]. Table 1 summarizes the hardness and Young's modulus for various BFO thin films obtained from different deposition methods and indentation operation modes.

**Table 1:** Hardness and Young's modulus of BFO thin films obtained from various deposition methods

	***H *(GPa)**	***E *(GPa)**
Radio frequency magnetron sputtering-derived BFO^a^		
350°C	6.8	131.4
400°C	8.5	147.6
450°C	10.6	170.8
Sol–gel-derived BFO [26]	2.8~3.8	26~51

^a^The present work.

**Figure 4:**
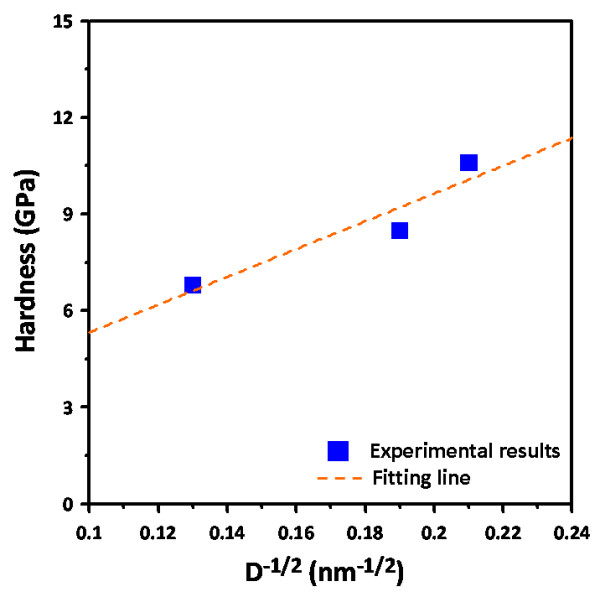
**Plot of the experimental data of hardness versus grain size.** The dashed line represents a fit to the Hall–Petch equation with *H*(*D*) = 1.03 + 43.12 *D*^−1/2^.

It is well known that the dependence of material hardness on the grain size can be described by the phenomenological ‘Hall-Petch’ equation [27]:

(5) $$H\left(D\right)=H_{0}+k_{\mathrm{H-P}}\;D^{-1/2}$$

where *H*_0_ and *k*_H-P_ are denoted as the lattice friction stress and the Hall–Petch constant, respectively. A plot of the hardness versus *D*^−1/2^data for BFO thin films deposited at various temperatures is displayed in Figure 4. We note that although the grain size of BFO thin films remains relatively small as compared to that of the usual metallic materials, the data still follow pretty closely to the Hall–Petch relation, and the so-called negative Hall–Petch effect [28] is not observed here. The dashed line represents the fit to the Hall–Petch equation for the experimental data, which gives

(6) $$H\left(D\right)=1.03+43.12\;D^{-1/2}$$

which indicates a probable lattice friction stress of 1.03 GPa, and the Hall–Petch constant of 43.12 GPa nm^1/2^ for BFO thin films also indicates the effectiveness of the grain boundary in hindering the dislocation movements.

Furthermore, it is evident that both the hardness and Young's modulus of BFO thin films decrease monotonically with increasing deposition temperature. The corresponding hardness values (Young's modulus) are 10.6 (170.8), 8.5 (147.6), and 6.8 (131.4) GPa for BFO thin films deposited at 350°C, 400°C, and 450°C, respectively. Since the higher deposition temperature leads to the larger grain size for BFO thin films, as we have discussed previously, it is reasonable to consider that the decrease of hardness and Young's modulus might be mainly due to the grain size effect [29].

## Conclusion

In conclusion, we have carried out the XRD, AFM, and nanoindentation techniques to investigate the fundamental nanomechanical properties and their correlations with the microstructural features of the technologically important multiferroic BFO thin films. The XRD analysis showed that BFO thin films were equiaxial polycrystalline in nature, albeit that the predominant (110) orientation and a rougher surface morphology were gradually developed with increasing deposition temperature. Nanoindentation results indicated that, depending on the grain size which is intimately related to the deposition temperature, BFO thin films have hardness ranging from 6.8 to 10.6 GPa and Young's modulus ranging from 131.4 to 170.8 GPa with the higher values corresponding to lower deposition temperatures. In addition, the hardness of BFO thin films appears to follow the Hall–Petch equation rather satisfactorily, and the Hall–Petch constant of 43.12 GPa nm^1/2^ suggests the effectiveness of grain boundary in inhibiting the dislocation movement in BFO thin films.

## Competing interests

The authors declare that they have no competing interests.

## Authors' information

SRJ is an associate professor and YCT is a designated topic student (in the Department of Materials Science and Engineering, I-Shou University, Kaohsiung, Taiwan). HWC is an associate professor and PHC is a master student (in the Department of Applied Physics, Tunghai University, Taichung, Taiwan). JYJ is a professor (in the Department of Electrophysics, National Chiao Tung University, Hsinchu, Taiwan).

## Authors’ contributions

SRJ designed the project of experiments; performed the XRD, AFM, and nanoindentation analyses; and drafted the manuscript. YCT dealt with the experimental data. HWC and PHC carried out the growth of BFO thin films, and JYJ participated in the paper discussion. All authors read and approved the final manuscript.
